# Inhaled gold nanoparticles cause cerebral edema and upregulate endothelial aquaporin 1 expression, involving caveolin 1 dependent repression of extracellular regulated protein kinase activity

**DOI:** 10.1186/s12989-019-0324-2

**Published:** 2019-10-16

**Authors:** Ching-Yi Chen, Po-Lin Liao, Chi-Hao Tsai, Yen-Ju Chan, Yu-Wen Cheng, Ling-Ling Hwang, Kuan-Hung Lin, Ting-Ling Yen, Ching-Hao Li

**Affiliations:** 1 Department of Physiology, School of Medicine, College of Medicine, Taipei Medical University, 250 Wuxing Street, Taipei, 110 Taiwan; 2 Graduate Institute of Medical Sciences, College of Medicine, Taipei Medical University, Taipei, Taiwan; 3 School of Pharmacy, Taipei Medical University, Taipei, Taiwan; 4 Institute of Food Safety and Health Risk Assessment, School of Pharmaceutical Sciences, National Yang-Ming University, Taipei, Taiwan; 5 Institute of Biomedical Sciences, Mackay Medical College, New Taipei city, Taiwan; 6 Department of Medical Research, Cathay General Hospital, Taipei, 22174 Taiwan

**Keywords:** Gold nanoparticle, Aquaporin 1, Endothelial cell, Caveolin 1, ERK, Edema

## Abstract

**Background:**

Gold nanoparticles (Au-NPs) have extensive applications in electronics and biomedicine, resulting in increased exposure and prompting safety concerns for human health. After absorption, nanoparticles enter circulation and effect endothelial cells. We previously showed that exposure to Au-NPs (40–50 nm) collapsed endothelial tight junctions and increased their paracellular permeability. Inhaled nanoparticles have gained significant attention due to their biodistribution in the brain; however, little is known regarding their role in cerebral edema. The present study investigated the expression of aquaporin 1 (AQP1) in the cerebral endothelial cell line, bEnd.3, stimulated by Au-NPs.

**Results:**

We found that treatment with Au-NPs induced AQP1 expression and increased endothelial permeability to water. Au-NP exposure rapidly boosted the phosphorylation levels of focal adhesion kinase (FAK) and AKT, increased the accumulation of caveolin 1 (Cav1), and reduced the activity of extracellular regulated protein kinases (ERK). The inhibition of AKT (GDC-0068) or FAK (PF-573228) not only rescued ERK activity but also prevented AQP1 induction, whereas Au-NP-mediated Cav1 accumulation remained unaltered. Neither these signaling molecules nor AQP1 expression responded to Au-NPs while Cav1 was silenced. Inhibition of ERK activity (U0126) remarkably enhanced Cav1 and AQP1 expression in bEnd.3 cells. These data demonstrate that Au-NP-mediated AQP1 induction is Cav1 dependent, but requires the repression on ERK activity. Mice receiving intranasally administered Au-NPs displayed cerebral edema, significantly augmented AQP1 protein levels; furthermore, mild focal lesions were observed in the cerebral parenchyma.

**Conclusions:**

These data suggest that the subacute exposure of nanoparticles might induce cerebral edema, involving the Cav1 dependent accumulation on endothelial AQP1.

## Background

Engineered nanomaterials are those that are intentionally produced in the size range of 1 to 100 nm. Nanomaterials usually exhibit distinct physicochemical properties that are different from their bulk form. In this sense, nanomaterials have potentially interesting applications in many fields. Gold nanoparticles (Au-NPs) have been enormously explored in electronics (sensors, solar cells or catalysis) and in biomedical applications (drug targeted delivery, molecular imaging, diagnosis and photothermal therapeutics) [1, 2]**.** Thus, human exposure to Au-NPs has been increasing, meanwhile, the safety concerns are gaining more attention.

Gold is an inert element and in bulk state is known to be highly compatible with human tissues. The ionic state of gold salts is reactive and has been involved in the treatment of rheumatoid arthritis for decades. As previously mentioned, either the particle size or the surface properties of Au-NPs make them potentially crucial in biodistribution, circulation and clearance from tissues, proving reactive to the human body [3]**.** For example, Au-NPs (2–5 nm) present various cytotoxic profiles [4]**.** After intravenous administration of Au-NPs with different diameters, smaller particles were found to infiltrate more organs than the larger ones [5]**.** Au-NPs (15–50 nm) can penetrate the blood-brain barrier (BBB), and accumulate heterogeneously throughout the brain, subjected to produce genotoxic events [6]**.** Our previous study demonstrated that Au-NPs caused endothelial paracellular leakage by altering components of endothelial tight junctions [7]**.** Therefore, the safety evaluation of Au-NPs does not directly translate from its bulk or ionic states. It is necessary to understand the potential hazard of Au-NPs to human health. In this study, we will elucidate the influence of Au-NPs on endothelial water transport.

Aquaporins (AQPs), are a family of proteins that have a basic function of water transport, ubiquitously found in cell membrane of animals and plants. In mammals, a total of 13 AQP subtypes, with the nomenclatures from AQP0 to AQP12, are known. Their appearances are cell-type dependent, wherein they play key roles in physiological balance of intracellular and intercellular fluid flow. Among these AQPs, AQP3, AQP7, AQP9, and AQP10 are aquaglyceroporins that transport both water and glycerol, whereas others are mainly water-selective channels [8]**.** The structure of AQP subtypes is highly conserved, each consisting of six transmembrane α-helical domains, that form the central pore for water movement. The functional unit of water channel is an assembly of four AQP monomers that osmotically regulate bidirectional movement of water [9, 10]**.**

The first discovered water channel protein, AQP1, is strongly expressed in epithelial cells of a number of tissues, especially in proximal tubules, descending thin limbs of the nephron, and glands [10, 11]**;** and in endothelial cells of most microvasculatures, including cornea, intestinal lacteals, endometrium [9, 10, 12, 13]**.** Furthermore, AQP1 is extensively expressed in proliferating microvessels of malignant tumors [14]**.** In these tissues, AQP1 facilitates water exchange, as well as the clearance of accumulating water from these cells. For example, in lung tissues, AQP1 and AQP5 are mainly found to express in capillary and alveolar epithelium, respectively [15]**.** An alternation in AQP1 protein expression may be mechanistically involved in the airway inflammation and chronic obstructive pulmonary disease generating a strong potential risk of pulmonary edema [16, 17]**.** Moreover, an induction of AQP1 either in brain, or in myocardial tissues is closely implicated with edema and development of various diseases [18–22]**.** Limited studies have been conducted exploring endothelial AQP1 and the exposure of nanoparticles. In the present study, using immortalized cerebral vascular endothelial cell line bEnd.3, we determined that Au-NP exposure made bEnd.3 cells more permeable to water by upregulating AQP1 expression, involving the caveolin 1 (Cav1) dependent repression on extracellular regulated protein kinases (ERK) activity. In vivo studies affirmed that inhaled Au-NPs boosted AQP1 expression and water content in cerebral tissues. Our findings provide an experimental basis to identify the effect of nanoparticles in brain injuries.

## Results

### Characteristics of gold particles

The particle size and particle size distribution of the gold particles were measured previously [7], and the data are summarized in Additional file 1: Table S1. The mean particle size of Au-NPs and Au-MPs was 40 ± 1 and 637 ± 9 nm, respectively.

### Au-NPs exerted no clear cytotoxicity on bEnd.3 cell

bEnd.3 cells were incubated with Au-NPs (or Au-MPs) for 24 h and then their viability was measured. In gold particle-treatment group, at concentrations up to 500 ng/mL, the level of vital cells (% relative to control) was 97.20 ± 13.60 (Au-NPs) and 100.87 ± 6.17 (Au-MPs), respectively. Treatment with Au-NPs (or Au-MPs) demonstrated no clear cytotoxicity on bEnd.3 cells **(**Additional file 1: Table S2).

### Au-NPs caused AQP1 accumulation in treated endothelial cells

**Fig. 1:**
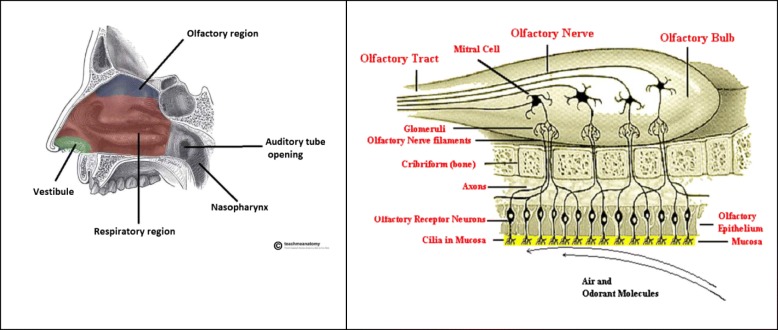
Au-NPs induced aquaporin-1 (AQP1) protein expression in bEnd.3 cells. **a**/**b** The bEnd.3 cells (an immortalized mouse cerebral endothelial cell line) were exposed to Au-NPs (or Au-MPs) and the expression level of AQP1 was detected by western blots. Representative images showed an increase of AQP1 protein level in Au-NP-treated groups, whereas AQP1 protein level remained unaffected in Au-MP-treated groups. **a** concentration-dependent treatment; cells were incubated with 10, 50, 100 and 500 ng/mL Au-NPs for 24 h. **b** time-dependent treatment; cells were incubated with 500 ng/mL Au-NPs for 3, 6, 12, and 24 h. (* *p* < 0.05, ** *p* < 0.01, and *** *p* < 0.001 indicates statistically significant difference from the control group; *N* = 11). **c** Representative images of immunofluorescent staining, the Au-NP-induced AQP1 and the nucleus was manifested by red and blue fluorescence, respectively. A gain of red fluorescence in cell membrane and cytosol was observed in Au-NP-treated bEnd.3 cells (500 ng/mL; 24 h), as compared to control. **d** Transendothelial permeability assay was performed as described in Materials and Methods. Au-NP treatment (500 ng/mL; 24 h) made bEnd.3 cell more permeable to water. (* *p* < 0.05, indicates statistically significant difference from the control group; *N* = 12)

The change in the expression levels of AQP1 was observed by western blotting. Treatment with Au-NPs (50, 100 and 500 ng/mL; 24 h) caused an induction of AQP1 protein levels in a concentration-dependent manner **(**Fig. 1a**)**. In time-course studies (500 ng/mL; 3, 6, 12, and 24 h), AQP1 began to accumulate, either at protein level **(**Fig. 1 b**)** or at mRNA level **(**Additional file 1: Figure S1). A significant induction of AQP1 protein was observed after 3 h of treatment, and this induction persisted for at least 24 h. The induction of AQP1 was also evidenced in Au-NP-treated HUVECs **(**Additional file 1: Figure S2). No significant changes were found in the level of AQP1 protein in Au-MP-treated groups. These data suggest that AQP1 induction is a response unique to Au-NPs and potentially leads to nanotoxicity that is distinctly different from bulk toxicity of substances.

Next, the location of AQP1 was observed by immunofluorescence staining. In control cells, the AQP1 expression signals were weak and sporadic in the whole cell. After Au-NP treatment (500 ng/mL; 24 h), the fluorescence intensity of AQP1 was enhanced, moreover, the distribution of AQP1 was detected in cytosol as well as cell membrane **(**Fig. 1 c**)**. The water permeability of bEnd.3 cells was measured by the diluent effects of the fluorescent probe TexasRed™-dextran. Briefly, cell layer was prepared in upper chamber of Transwell (0.4 μm pore size), then the medium in upper chamber was replaced by hypertonic PBS (with 300 mM D-mannitol and TexasRed-dextran) before testing. No significant water flux from the lower chamber to upper chamber was observed in control group; by contrast, in cells treated with Au-NPs (500 ng/mL; 24 h), a decrease in fluorescence intensity of TexasRed-dextran was observed after 10–30 min **(**Fig. 1 d**)**, which suggested that Au-NP-treated bEnd.3 cells became more permeable to water.

### Au-NPs increased focal adhesion kinase (FAK) and AKT phosphorylation but prevented phosphorylation of extracellular signal regulated kinases (ERK), contributing to Au-NP-mediated AQP1 accumulation

**Fig. 2:**
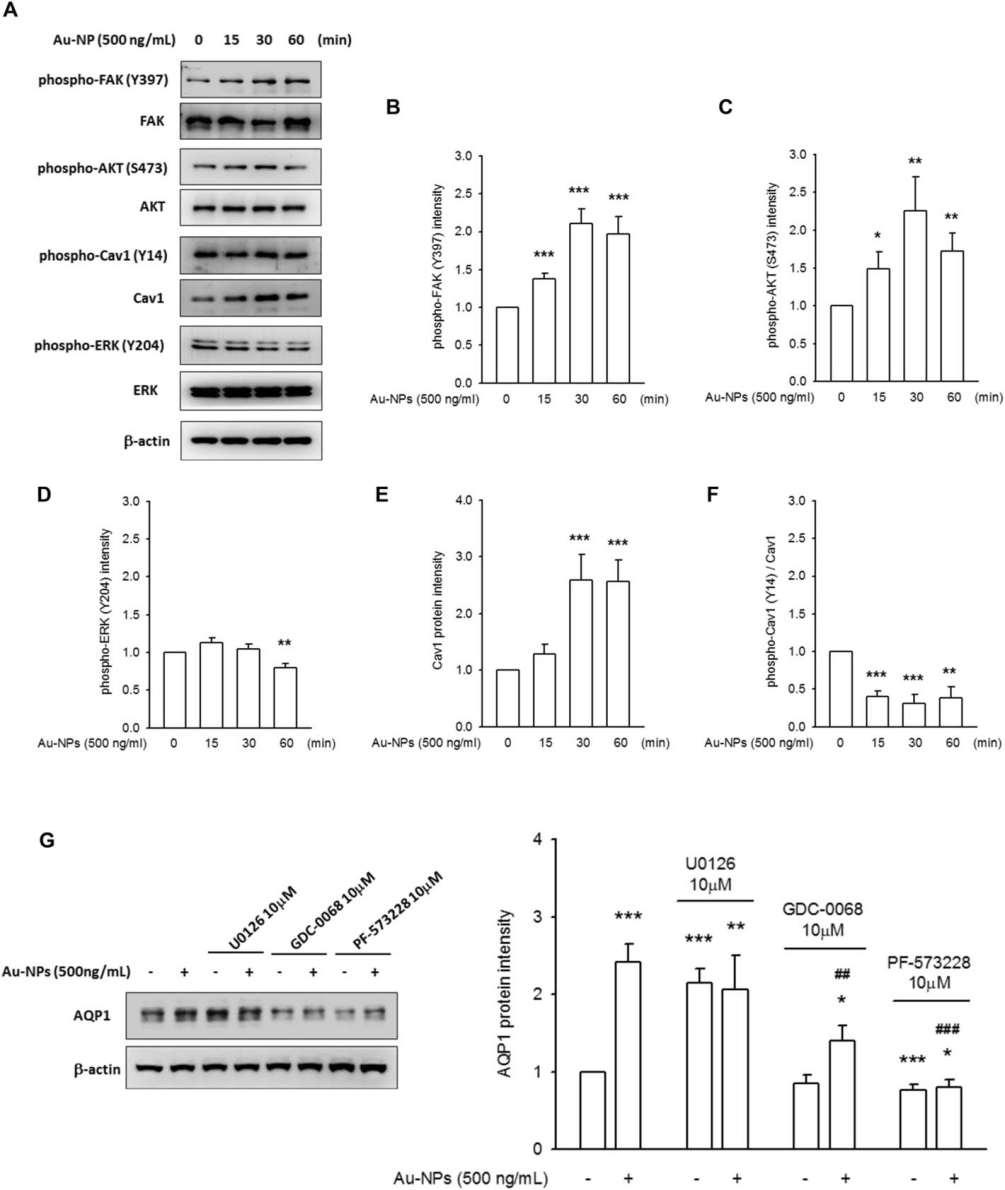
Au-NP treatment rapidly changed the phosphorylating status of FAK, AKT and ERK, and subsequently contributing to Au-NP-mediated AQP1 accumulation. bEnd.3 cells were incubated with 500 ng/mL Au-NPs for 15, 30 and 60 min, and the phosphorylating status of FAK, AKT, ERK, and Cav1 was measured by western blots. **a** Representative images showed an augmentation of FAK and AKT phosphorylation; a reduction of ERK and Cav1 phosphorylation in Au-NP-treated groups in time-dependent manner, as compared to control. Also, Au-NP treatment caused an accumulation of Cav1 protein level. Quantified data was gained by densitometry analysis, followed by a normalized process to their total form. **b** phospho-FAK, (**c**) phospho-AKT, (**d**) phospho-ERK, (**e**) Cav1 and (**f**) phospho-Cav1. (* *p* < 0.05, ** *p* < 0.01, and *** *p* < 0.001 indicates statistically significant difference from the control group; *N* > 10). **g** Cells were pre-incubated with 10 μM U0126 (ERK inhibitor), 10 μM GDC-0068 (pan-AKT inhibitor) and 10 μM PF-573228 (FAK inhibitor), subjected to a 12–16 h exposure of Au-NPs. Images and quantified data revealed that Au-NP-induced AQP1 expression was prevented while FAK and AKT inhibition, whereas an enhancement of AQP1 expression was presented in the presence of U0126. (* *p* < 0.05, ** *p* < 0.01, and *** *p* < 0.001 indicates statistically significant difference from the control group; ## *p* < 0.01, and ### *p* < 0.0001 indicates statistically significant difference from the Au-NP-treated group; *N* = 7)

The activation of Cav1-AKT-ERK pathway, found in hydrostatic pressure-treated A549, has been proved in the augmentation of AQP1 expression [23]**.** But, in this study, after 15–60 min incubation of 500 mg/mL Au-NPs, the levels of the phosphorylated form of FAK (Tyr-397) and AKT (Ser-473) were enhanced in time-dependent manner, whereas the phosphorylation levels of Cav1 (Y-14) were not unaffected. The phosphorylation of EKR was reduced after 60 min of Au-NP incubation. Interestingly, the amount of Cav1 was increased after 30–60 min incubation with Au-NPs, resulting in an obvious reduction of phospho-Cav1/total Cav1 ratio **(**Fig. 2 a-f**)**. Au-NP-mediated AKT and FAK phosphorylation, ERK de-phosphorylation and Cav1 induction were observed in a concentration-dependent fashion **(**Additional file 1: Figure S3).

Pretreatment with 10 μM GDC-0068 (a highly selective pan-Akt inhibitor) and 10 μM PF 573228 (FAK inhibitor) caused a pretty prevention on Au-NP-mediated AQP1 induction **(**Fig. 2 g**)**. While ERK inhibition by 10 μM U0126, a significant accumulation of AQP1 protein was existed, regardless of the presence of Au-NPs **(**Additional file 1: Figure S4). These data suggest a positive regulation of FAK and AKT signaling in Au-NP-mediated AQP1 expression; whereas ERK induced negative regulation of AQP1.

### Caveolin-1 (Cav1) is a crucial factor in Au-NP-mediated AQP1 expression

**Fig. 3:**
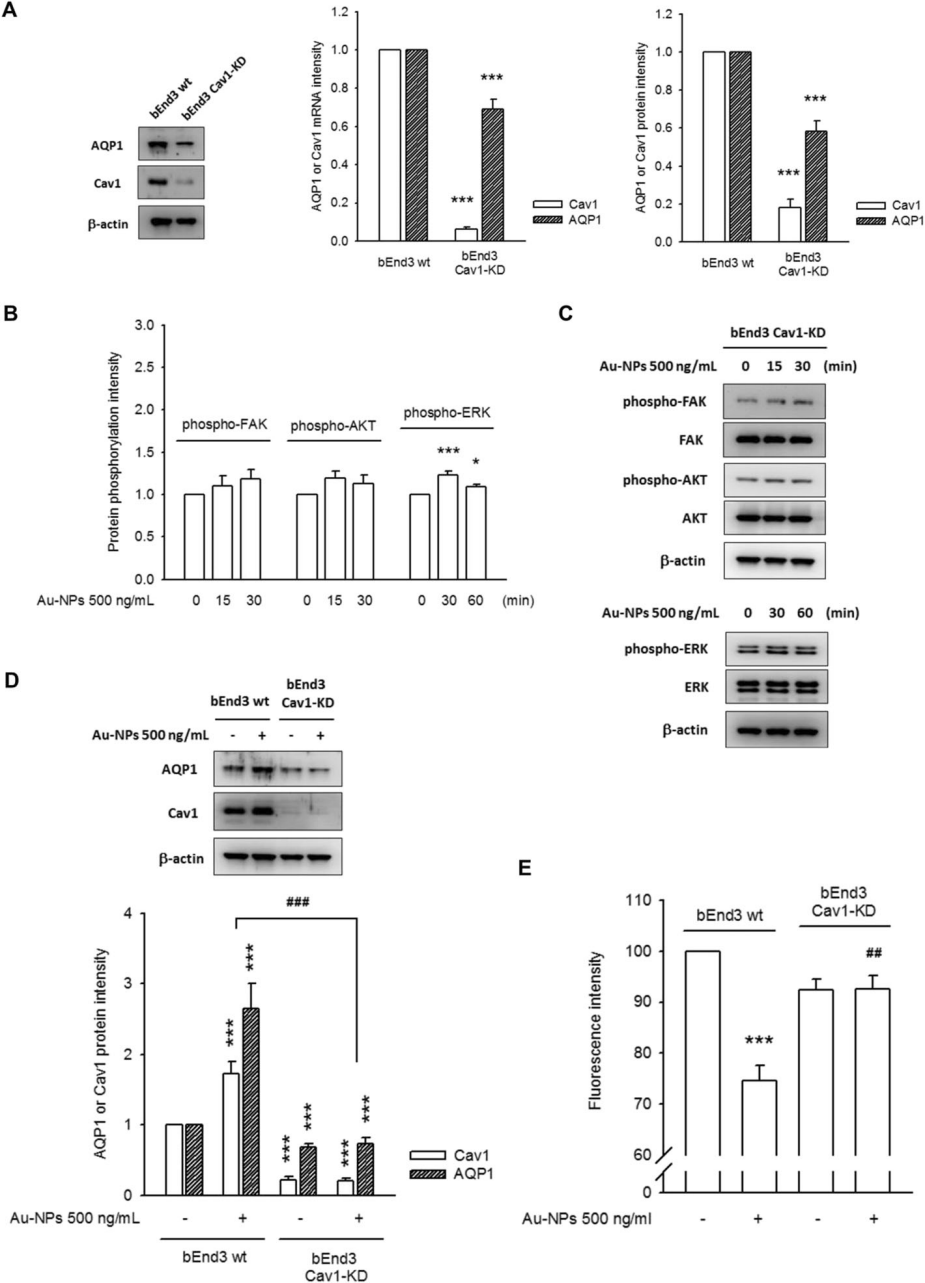
Caveolin-1 (Cav1) is a crucial factor in Au-NP-mediated AQP1 expression. **a** bEnd.3 cells with a silenced Cav1 (bEnd.3 Cav1-KD) was established as described in Materials and Methods. The mRNA and protein expression level of Cav1 and AQP1 was obviously down-regulated. **b**/**c** Both images and quantitative data proved Au-NP-mediated FAK and AKT phosphorylation (as well as ERK de-phosphorylation) were prevented in bEnd.3 Cav1-KD (*N* = 6). **d** Au-NP-mediated AQP1 induction and (**e**) water permeability was also ameliorated in bEnd.3 Cav1-KD, as compared to wild-type (N = 6). These data manifested the requirement of Cav1 of bEnd.3 cell in respond to Au-NP stimulation. (* *p* < 0.05, ** *p* < 0.01, and *** *p* < 0.001 indicates statistically significant difference from the control group; ## *p* < 0.01, and ### *p* < 0.001 indicates statistically significant difference from the Au-NP-treated group)

Vascular endothelial cells have an abundance of Cav1 protein, which functions as the key constituent of caveolae and recruited multiple signaling factors in caveolae [24]**.** Au-NPs caused a rapid accumulation of Cav1 in treated bEnd.3 cells, parallel to AQP1 accumulation. Importantly, Au-NP-induced AQP1 induction was not observed in those without or with restricted Cav1 expression (HEK293T, EA.hy929 and BEAS-2B) **(**Additional file 1: Figure S2C-S2D). To identify the role of Cav1 response to Au-NPs administration, we created bEnd.3 stable clones, Cav1-KD, with a silenced Cav1 expression. The basal expression levels of Cav1 and AQP1 was downregulated in bEnd.3 Cav1-KD, either at the transcriptional or at the translational level **(**Fig. 3a**)**. Moreover, the FAK, AKT and ERK of bEnd.3 Cav1-KD did not respond to Au-NP challenge **(**Fig. 3 b**)**, as a consequence, neither Au-NP-induced AQP1 induction nor Au-NP-mediated water permeability was observed in bEnd.3 Cav1-KD, as compared to bEnd.3 wt **(**Fig. 3 c, d**)**.

**Fig. 4:**
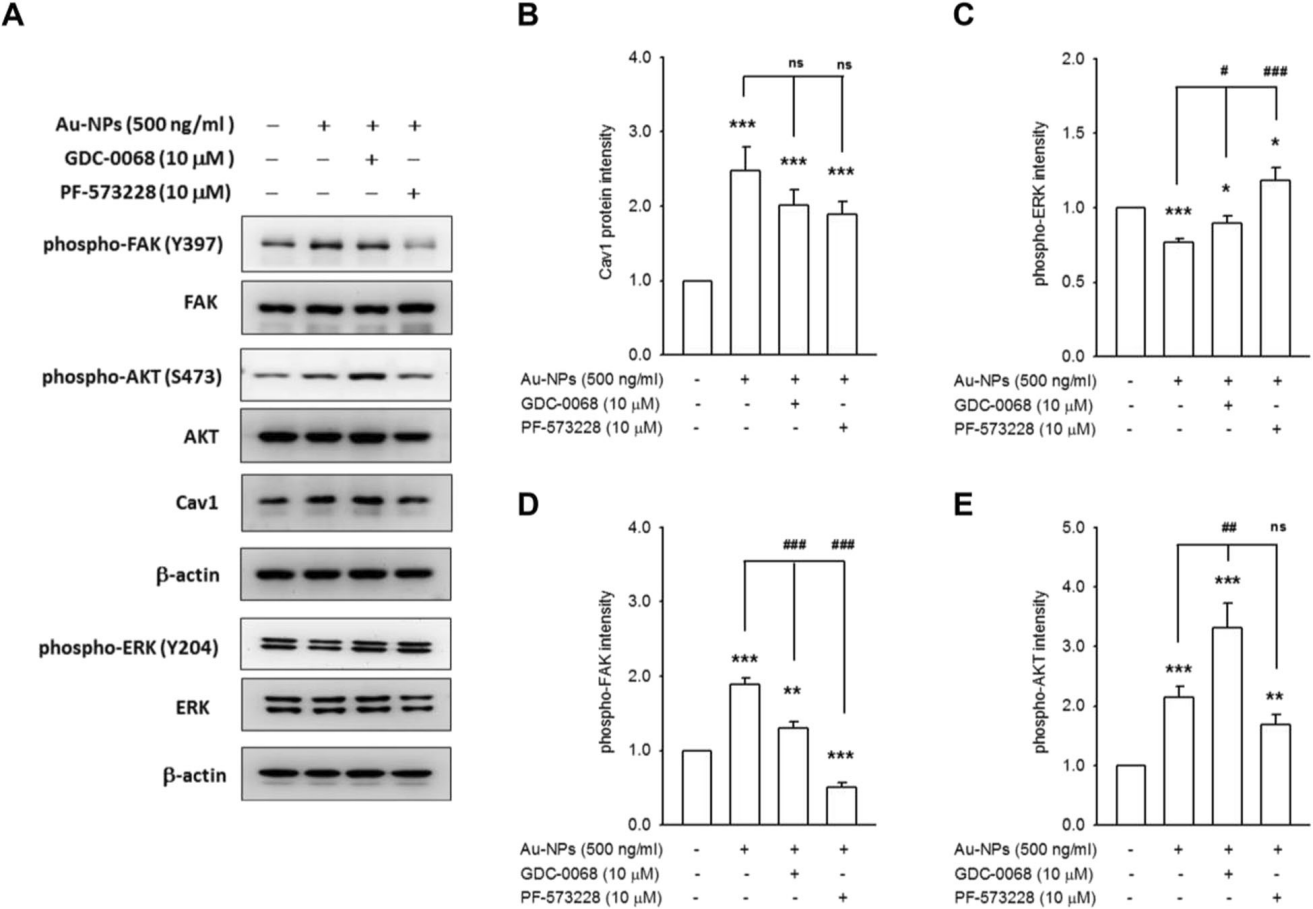
Cav1 is of central importance to transduce signal after Au-NP exposure. Representative images (**a**) and quantitative data proved 10 μM GDC-0068 and 10 μM PF-573228 has no obvious influence on Au-NP-mediated Cav1 accumulation (**b**), but significantly reversed Au-NP-mediated ERK de-phosphorylation (**c**). PF-573228 decreased the phosphorylation of FAK and AKT induced by Au-NPs, suggested FAK may be an upstream effector (**d**). GDC-0068 caused a robust phospho-AKT, which is the signature of ATP competitive inhibitors of AKT, but failed to prevent Au-NP-induced FAK phosphorylation (**e**). (* *p* < 0.05, ** *p* < 0.01, and *** *p* < 0.001 indicates statistically significant difference from the control group; # *p* < 0.05, ## *p* < 0.01, and ### *p* < 0.001 indicates statistically significant difference from the Au-NP-treated group. The ns means without statistical difference.)

Cav1 is also known to interact with a number of signaling molecules in transduction of extracellular mechanical stimuli [24]**.** Pretreatment with GDC-0068 or PF-573228 did not affect Au-NP-mediated Cav1 accumulation but decreased the phosphorylation of FAK and AKT induced by Au-NPs. The robust phospho-AKT in GDC-0068 treated group was the result of the binding of the ATP competitive inhibitors to the active site of AKT, wherein they also protected these sites from phosphatases [25]**.** Both GDC-0068 and PF-573228 rescued Au-NP-mediated ERK de-phosphorylation **(**Fig. 4**)**. These data suggest the Cav1 is of central importance to transduce the signal from Au-NP exposure, and consecutive up-regulation of AQP1.

### Inhaled Au-NPs increased water permeability in vivo

**Fig. 5:**
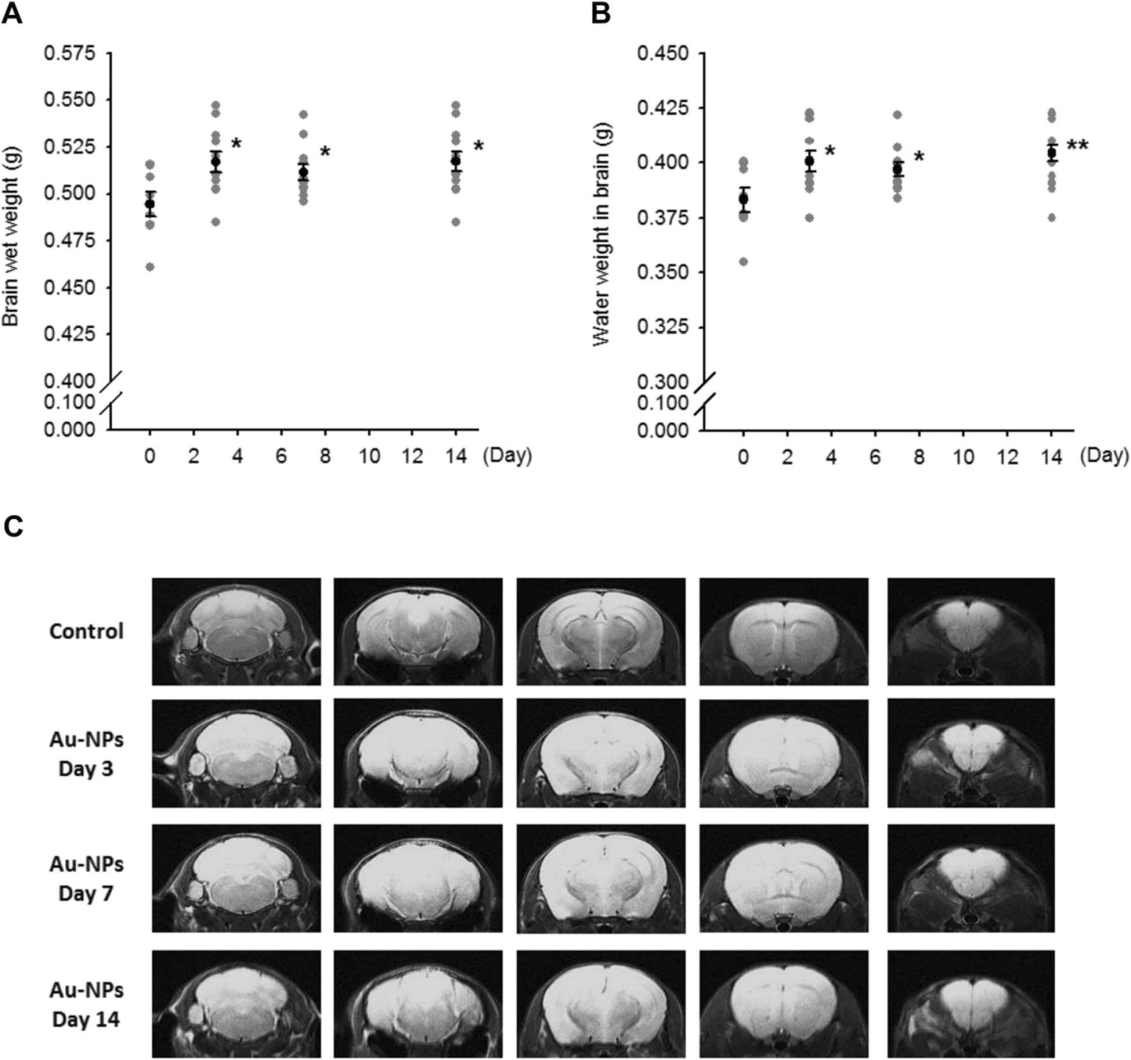
Inhaled Au-NPs increased water permeability in vivo. After intranasal Au-NP administration (3 mg/kg) for 3–14 days, ICR mice were gained in their brain wet weight (**a**), as well as the water content in brain (**b**). (* *p* < 0.05, and ** *p* < 0.01 indicates statistically significant difference from the control group, *N* = 8). By using non-invasive method, Au-NP-mediated brain edema has been affirmed in T2 MRI scanning. An elevation in T2 value was detected in day 3–14, as compared to control animals

There were no changes in behaviors or in the numbers of moribund or dead animals related to intranasal Au-NP administration. Animals were sacrificed at 0 (control group), 3, 7, and 14 days post-dosing, and the weight of brains, either the fresh, wet tissues or the frozen-dried tissues, were recorded. Data showed significant increase in brain wet weight in Au-NP-treated groups **(**Fig. 5a**)**, as compared to control group; additionally, the amount of water in the brain was also augmented **(**Fig. 5 b**)**. Next, inhaled Au-NPs induced brain edema was authenticated by T2 MRI. In Fig. 5 c, MRI images across a series section of brain showed T2 signals were elevated after 3 days (incidence 2/7) and remained elevated on day 7 (4/6) and 14 (5/7). The hyperintensity T2 signal represented an accumulation of water in whole brain tissues of Au-NP-treated mice.

### Inhaled Au-NPs upregulated AQP1 in cerebral microvessels in vivo

**Fig. 6:**
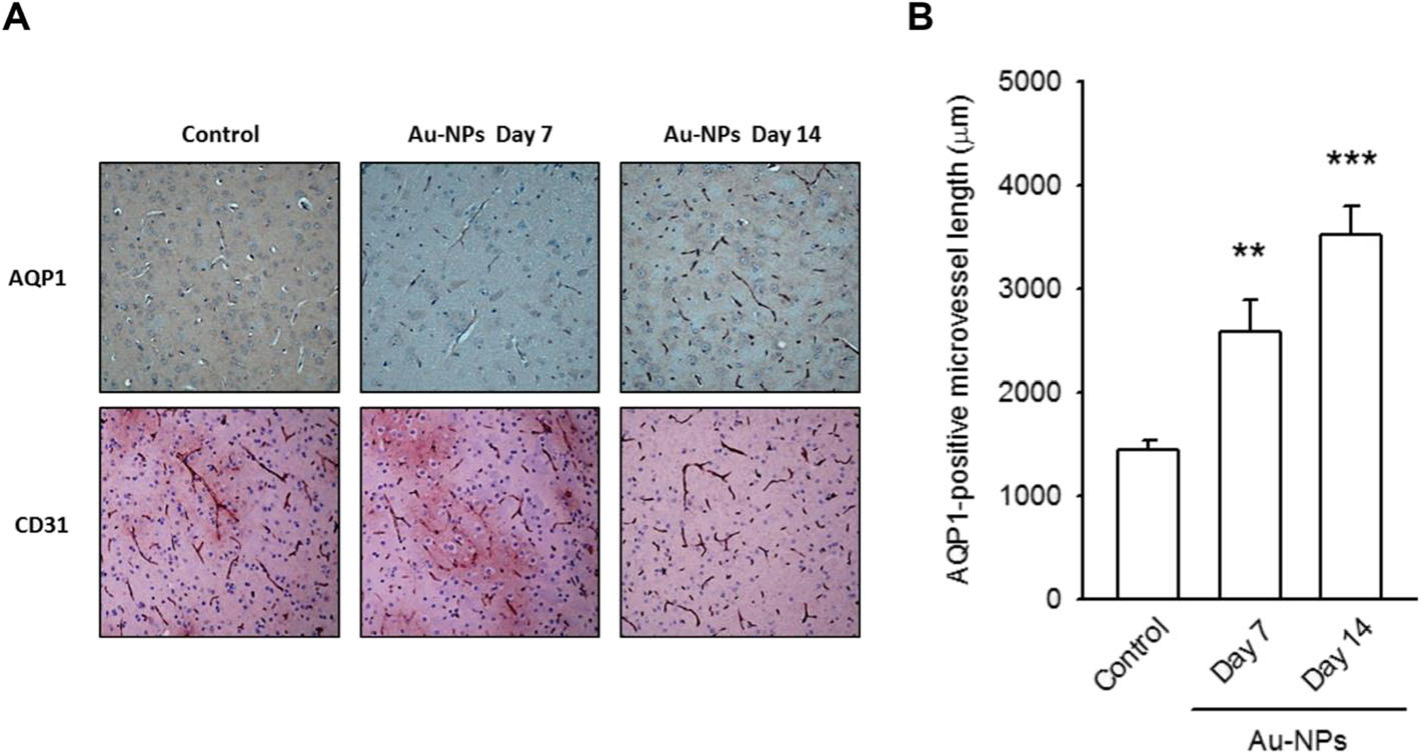
Inhaled Au-NPs upregulated AQP1 expression in cerebral microvessels in vivo. **a** Representative images showed an increase in immunoreactivity against AQP1 in microvessels of brain tissues, whereas the stained CD31 (an endothelial biomarker) was without significant differences. **b** The quantitative data was determined by the measurement of the length of AQP1-positive microvessels. (** *p* < 0.01, and *** *p* < 0.001 indicates statistically significant difference from the control group, *N* = 4)

The immunohistochemical staining of brain slices was performed with antibodies against AQP1 and CD31. Compared to the immunoreactivity in control tissue, a significantly increased vascular expression of AQP1 could be detected, especially the region around the diencephalon, after a 7-day or 14-day intranasal Au-NP administration. The immunostaining for CD31 was continuously positive in microvessels of cerebral cortex **(**Fig. 6a**)**. The corresponding quantitative data is shown in Fig. 6 b.

### Inhaled Au-NPs caused mild tissue damages in brain

**Fig. 7:**
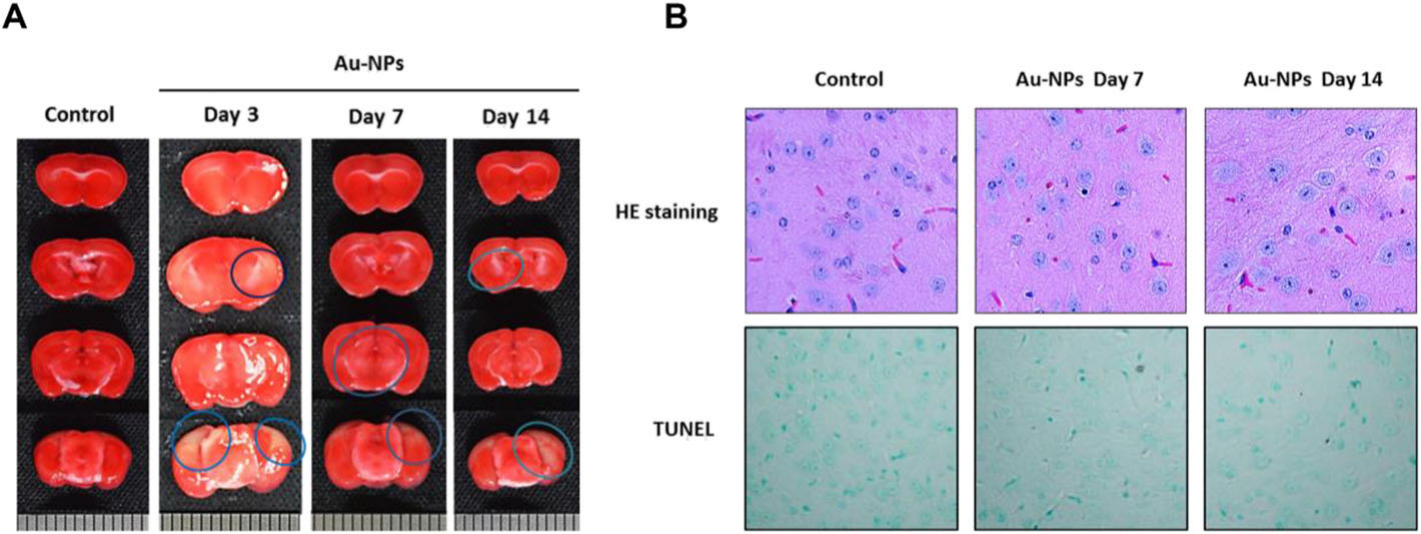
Inhaled Au-NPs caused mild tissue damages in the brain. **a** Briefly, brain slices were made freshly, and incubated with 2% TTC solution as described in Materials and Methods. Representative images showed in Au-NP-treated groups, the reactivity of brain tissues to metabolize TTC to red-colored, lipid-soluble formazan were decreased, suggested the appearance of mild, focal tissue damages. **b** Histopathological lesions of brain were accessed by H&E staining and TUNEL assay. Samples of Au-NP-treated groups had a weak reactivity to eosin, and the morphology of neurons are swelling with a finely, adjacent vacuolated region around the cell body. However, no darkly stained TUNEL-positive cells were found

Histological lesions of brain were detected by TTC staining. Representative images of TTC-stained brain sections are shown in Fig. 7a. We found that the cerebral cortex and the diencephalon region of mice receiving 3–14 days Au-NP administration had a lower reactivity to TTC metabolism, which showed a pink color rather than dark red color, as compared to control group. Furthermore, histological damage of brain tissue was accessed by hematoxylin and eosin staining and TdT-mediated-dUTP nick end labeling (TUNEL) assay. We found the brain tissues of mice receiving 7- and 14-day Au-NP treatment had a weak affinity to eosin. In diencephalon region of Au-NP-treated groups, the stained neurons were swollen in appearance with a finely, adjacent vacuolated region around the cell body. However, no TUNEL-positive cells were found **(**Fig. 7 b**)**. These changes are frequently associated with the pathogenic signs of cerebral edema.

## Discussion

The balance of water influx and efflux is of central importance to maintain the functioning and hemostasis of our body. Generally, fluid and solute transport is executed by two pathways [26]**.** In paracellular pathway, water moves through the intercellular spaces. A number of inflammatory mediators targeting endothelial junctions, resulted in the opening of paracellular gaps [27]**.** The transcellular pathway occurs in response to osmotic tones, consequently by the trafficking and synthesis of AQPs, the water flows rapidly across both basal and apical surface of cells. In this study, we primarily demonstrated that exposure to Au-NPs made endothelial cells permeable to water through the upregulation of AQP1 expression.

The pathophysiological role of AQP1 has been established in AQP1^−/−^ animals. The development in these animals has been observed to progress normally. However, they display profound abnormalities associated with transcellular water transport across a variety of organs [11, 28, 29]**.** Also, induction of myocardial infracts in AQP1^−/−^ mice revealed a significantly reduced myocardial edema [30]**.** These data support the role of AQP1 as the chief mediator in edema formation. In this study, intranasal Au-NP exposure for 3–14 days led to AQP1 induction in microvessels or water accumulation in brain tissues of treated mice. This hints at the nose-to-brain delivery of nanoparticles, which can be correlated with Au-NP-mediated AQP1 expression and cerebral edema. The nose-to-brain delivery tract has been discussed as an alternative dosing route to bypass the blood-brain barrier (BBB). Briefly, after intranasal exposure, the nanoparticles (or chemicals, even cells) can be passively moved along the perineural space of olfactory nerves. Subsequently, these cross the olfactory epithelium, cribriform plate, and olfactory bulb, and eventually reach the diencephalon region and the olfactory cortex (the extracellular route). Alternatively, inhaled nanoparticles are internalized into endocytic vesicles of olfactory and trigeminal nerves, followed by axonal transport into the brain (the intracellular route) [31]**.** For example, after an intranasal colloidal silver spray, Ag-NPs have been detected within the olfactory epithelium with subsequent transport to the brain [32]**.** In the past few years, extensive research has been carried out on this aspect, which has improved the efficiency of brain targeting [31]**.**

An abundant expression of AQP1 has been characterized in microvasculature endothelial cells throughout the body, with an exception of the brain, where the water fluidity is under restricted control. In brains, AQP1 was primarily described in the epithelium of choroid plexus and thought to be required for cerebrospinal fluid production [28]**.** A limited AQP1 was expressed in capillary endothelial cells of the BBB, where the endothelium is tightly adhered and not fenestrated. It is intriguing that the primary cultures of these endothelial cells began to exhibit increase in their AQP1 expression levels during passages, but the AQP1 expression levels immediately reduce when co-cultured with astrocytes. That means the endothelial cells of the BBB retain their AQP1-expressing capacity, but robustly suppress it due to the close contact with perivascular astrocytes [33]**.** More importantly, the AQP1 expression has been detected in microvascular endothelium of circumventricular organs (CVOs). The CVOs are specialized brain regions localized in diencephalon region adjacent to the brain ventricles. They are devoid of the usual endothelial tight-junction appositions, instead of the fenestrated vasculature (also termed the windows on the brain). Among seven CVOs found in the brain, with exceptions of the sub-commissural organ and the vascular organ of the lamina terminalis, the microvessels of median eminence, pineal, subfornical organ, area postrema and choroid plexus are fenestrated. Most of their endothelial cells, if not all, are extensively AQP1 immunoreactive, allowing the highly permeable vessels (as compare with BBB vessels) to regularly modulate the fluid homeostasis and endocrine secretion. AQP1-positive endothelia were also detected in a small population of vessels in cerebral parenchyma [34–36]**.** In our study, either Au-NP-mediated AQP1 induction in microvessels or water accumulation was observed throughout the brain tissue, especially in the diencephalon region of mice receiving intranasally administered Au-NPs.

In this study, we also demonstrated Au-NP-mediated AQP1 expression is dependent on the expression levels of Cav1. Caveolins are essential transmembrane scaffolding protein for plasma membrane invagination and caveolae formation. There are 3 isoforms. The Cav1 and Cav2 are extensively co-expressed in many cell types (especially in endothelial cells), whereas Cav3 is expressed only in striated muscle cells. Among them, Cav1 has been well-characterized to serve as scaffolds to concentrate specific lipids and a number of signaling molecules, allowing the caveolae compartment to become more sensitive to mechanical and ligand-based stimuli [24, 37]**.** In this work, Au-NP treatment caused a rapid Cav1 protein accumulation, FAK and AKT phosphorylation and ERK de-phosphorylation in treated bEnd.3 cells. Pretreatment with GDC-0068 and PF-573228 had no deleterious effect on Au-NP-mediated Cav1 expression, but it restored ERK activity and prevented Au-NP-mediated AQP1 expression. We also found the accumulation of Cav1 and AQP1 in U0126 treated groups, indicating negative regulation of ERK in endothelial AQP1 expression. Moreover, by using bEnd.3 Cav1-KD cells, we found none of these signaling molecules, nor AQP1 expression in response to Au-NP treatment **(**Additional file 1: Figure S5). In other reports, genetic deletion of Cav1 in vivo resulted an elevated pulmonary microvascular permeability [38], the detrusor hypo-contraction and an impaired urinary bladder function [39, 40]**.** Both of them closely implicated the Cav1-dependent AQP1 regulation in microvasculature of the lung and urethra, respectively. Taken together, the Cav1 showed strong correlation to AQP1, either in expression or in function.

Although ERK is a well-established signal factor, sequestered within caveolae [41], the causation between ERK activity and Cav1 expression is still controversial and appears to be cell-type dependent. In astrocytes, Cav1 has been found to directly interact with Ras, subsequently promoting the phosphorylation of ERK, which is a pivotal signal for cell proliferation [42]**.** Hypoxic astrocytes manifested a remarkable reduction of Cav1, a diminished phosphorylation level of ERK, and an aggravated hypoxia-induced astrocyte injury [43]**.** In contrast, supplement of Cav1 peptide (the caveolin-scaffolding domain or CSD) not only heightened ERK phosphorylation levels, but also protected astrocytes from hypoxia-induced injuries. The protective effect of CSD was remarkably counteracted by U0126 [44]**.** Another report indicated that the protein interaction of Cav1 and kinase suppressor of Ras 1 (KSR1) is essential for ERK activation in mouse embryo fibroblasts (MEFs), and the disruption of Cav1- KSR1 interaction by KSR1 mutant evidently abolished ERK activation [45]**.** These data report Cav1 to be a positive regulator of ERK signaling in astrocytes and MEFs [46], however, lots of studies report the relationship between ERK activity and Cav1 exhibit reciprocally negative action in endothelial cells and macrophages [47, 48]**.** Connexin 43 inhibition in cultured HUVECs decreased the phosphorylation of ERK but increased the expression of Cav1. Chemical inhibition of the ERK pathway with PD-98059 upregulated Cav1 expression; by contrast, Cav1 siRNA downregulated ERK activity [49]**.** An abrogated ERK signaling found in endothelial cells derived from Cav^−/−^ mice could be restored while Cav1 transfection, but the ERK failed to activate in cells with high Cav1 expression levels [50]**.** These data aligned with our findings that the elevation of Cav1 protein levels dramatically inhibits ERK phosphorylation in Au-NP-treated endothelial cells. This may due to the direct protein interaction of ERK with Cav1 (unpublished data). The inhibitory domain of Cav1 has already been mapped and the synthetic peptides of Cav1 residues 32–95 could potentially inhibit purified ERK activity in vitro. On the contrary, constitutive activation of the ERK is sufficient to suppress Cav1 mRNA and protein expression [51, 52]**.**

Among FAK, AKT and ERK, ERK activity seemed more influential on AQP1 expression across cell types, either in a positive or in a negative manner. In astrocytes, Schwann cells and epithelial cells, activated ERK is required for the induction of AQP1 expression, whereas the induced AQP1 was significantly attenuated by the treatment with ERK inhibitor [53–56]**.** In endothelial cells, however, copper chelator treatment inhibited endothelial proliferation and migration, involving an increased phospho-ERK, but downregulated AQP1 [57]**.** Ultraviolet radiation, H_2_O_2_ and pre-B cell colony-enhancing factor (PBEF) have been shown to reduce AQP1 expression levels and enhance inflammatory stress. After blocking the ERK pathway, the downregulation of AQP1 was rescued [58, 59]**.** Herein, we proved the Au-NPs inhibited ERK activity, and ERK negatively regulated endothelial AQP1 expression.

## Conclusion

Nonetheless, this is the first study to demonstrate the role of inhaled Au-NPs in causing brain edema in vivo, involving Au-NP-mediated AQP1 induction in the endothelial cells of brain tissues. In addition, we showed that Au-NP-mediated endothelial AQP1 expression is Cav1 dependent through repression on ERK activity. Our findings not only corroborate with the human safety concerns in nanoparticles exposure, but also partially illustrate the role of particulate air pollution in the development of neurological disorders. In a more recent study, chronic inhalation of traffic-related particulate matter (PM1) has been identified to cause cerebral edema, spongiosis, and neuronal shrinking in adult rats [60]**.** In view of this, by modifying the expression or function of AQP1, may be significant in the development of protective strategies or therapeutic agents focusing on a range of brain pathologies.

## Materials and methods

### Cell culture

The immortalized mouse cerebral vascular endothelial cell line, bEnd.3, was purchased from Bioresource Collection and Research Center (BCRC, Hsinchu, Taiwan). The cells were routinely propagated using Dulbecco’s modified Eagle’s medium (DMEM) supplemented with 10% FBS and maintained at 37 °C in an incubator with humidified atmosphere containing 5% CO_2_. Stock cells were passaged twice every week.

### Determination of cell viability

The cytotoxic potential of Au-NPs was assayed by the conversion of 3-(4,5-dimethyl-2-thiazolyl)-2,5-diphenyl-2H-tetrazolium bromide (MTT) to insoluble formazan [61]**.**

### Western blotting

bEnd.3 cells grown on 60-mm dishes treated with Au-NPs at prescribed periods, for example, 3~24 h for AQP1 protein analysis and 10~60 min for kinase phosphorylation analysis. After treatment, cell whole lysates were prepared as described [61]**.** Protein samples were separated by SDS-PAGE and electro transferred onto PVDF membranes. The membranes were blocked using TBST containing 5% skim milk, and incubated overnight at 4 °C with primary antibodies against AQP1, phospho-Akt, FAK (CUSABIO, Houston, TX), Cav1, phospho-Cav1, phospho-FAK (Abcam, Cambridge, UK), Akt, ERK, phospho-ERK (Santa Cruz, Dallas, TX), and β-actin (Sigma-Aldrich, Chicago, IL). The membranes were then washed in TBST and incubated for 2 h at room temperature with horseradish peroxidase-conjugated secondary IgG (1:5000). Finally, blots were developed using enhanced chemiluminescence (ECL), and images were captured and densitometrically analyzed using the BioSpectrum AC® Imaging system (UVP, Upland, CA).

### Immunofluorescent staining

bEnd.3 cells were grown on glass cover slips. After Au-NP treatment (500 ng/mL; 24 h), the cells were fixed in 4% paraformaldehyde, permeabilized with 0.1% Triton X-100 for 5 min at room temperature, and incubated with blocking serum for 30 min. Next, the cells were incubated overnight (4 °C) with a primary AQP1 antibody (1:50; Bioss Antibodies, Woburn, MA, catalog. bs-1506R) and then with a fluorescein isothiocyanate (FITC)-conjugated secondary antibody (1:1000) at room temperature for 1 h in the dark. Finally, nuclei were counterstained with 5 μM Hoechst 33342 (Molecular Probes®, Eugene, OR) for 5 min and the samples were washed thrice and then mounted on slides. Fluorescent images were captured using a Leica TCS SP5 Confocal Spectral Microscope Imaging System.

### Transendothelial water permeability

Osmotic water permeability across endothelial cell layer was determined using Texas Red™-dextran (10 kDa; Molecular Probes, Eugene, OR) dilution method [62]**.** bEnd.3 cells were seeded at 1 × 10^5^ cells per insert in Transwell inserts with 0.4 μm pore size (Falcon, catalog No. 353095) and incubated for 2–4 days until cell layer formation. After Au-NP treatment (500 ng/mL; 24 h), the basal and apical surface of cells was bathed in isosmolar PBS and hyperosmolar PBS (PBS with 300 mM D-mannitol) containing 0.25 mg/mL Texas Red-dextran, respectively. Cultures were placed in incubator (37 °C, 5% CO_2_), and 5 μl samples of dye-containing apical fluid were collected at 10, 20 and 60 min. The samples were diluted in PBS, and fluorescence was measured by Fluoroskan Ascent™ microplate fluorometer (Thermo Scientific™, Vantaa, Finland).

### Reverse transcription and quantitative polymerase chain reaction (RT-qPCR)

Total RNA (3 μg), isolated by Trizol reagent (Invitrogen, Waltham, MA), was converted to complementary DNA by using MMLV reverse transcriptase (BioGenesis, Taiwan). The AQP1 and Cav1 fragments of complementary DNA were amplified and simultaneously quantified by LightCycler®Nano machine (Roche Molecular Systems, Inc., Almere, Flevoland, Nederland) and the OmicsGreen qPCR Master Mix reagent (Omicsbio, Taiwan). Relative levels of gene expression were quantified by a ΔΔCq calculation method. mRNA levels were normalized with β-actin. The primer sets of β-actin, AQP1, and Cav1 are listed in Additional file 1: Table S3.

### Animal husbandry and treatment

Male ICR mice (6–8 week old) purchased from AAALAC authenticated supplier (BioLASCO Co., Ltd., Taiwan) were grouped and acclimated for one week in the Animal Center of Taipei Medical University (Taipei, Taiwan). Mice in the test groups received Au-NPs by intranasal administration (3 mg/kg); mice in the negative control group received the vehicle (saline). The mortality and clinical behavior of the animals were observed daily. The animals were sacrificed as scheduled (0, 3, 7 and 14 days). The brain tissue was harvested and weighed immediately (wet weight). These were then freeze-dried, for measurement of dry weight. Brain water content was calculated using following formula: wet weight-dry weight.

### Magnetic resonance imaging (MRI)

On day 0, and day 3, 7 and 14 after Au-NP treatment, mice were anesthetized and placed in a carriage equipped with a stereotaxic holder, an integrated heating system to maintain the body temperature, and a detector to monitor the respiration. The head was taped to the carriage to minimize motion artifact. MRI images were captured on a 7-T/40-cm magnet, a Biospec Bruker console (Billerica, MA, USA), and a 40-G/cm gradient insert (ID = 12 cm, 120 μs rise time). A surface coil was used for brain imaging and a neck coil for perfusion labeling. Coil-to-coil electromagnetic interaction was actively decoupled. T2-weighted images were acquired using fast spin-echo pulse sequence with 2 effective echo times (50 and 80 msec), TR = 2 s (90 flip angle), matrix = 128 × 128, FOV = 2.56 × 2.56 cm, echo train length 8, and 8 signal averages.

### Immunohistochemistry

Isolated brain was immediately fixed in 10% phosphate-buffered formalin, subjected to routine procedure of paraffin wax embedding. A series of adjacent slices (5 μm thick) were made and then stained with hematoxylin and eosin for histopathology. To survey the expression of AQP1, brain slices were de-paraffinized and rehydrated. After antigen retrieval and non-specific antigen masking, the specimens were incubated with primary antibodies (anti-AQP1, Bioss Antibodies, Woburn, MA, Catalog bs-1506R; and anti-CD31, Abcam, Cambridge, UK, Catalog ab-182,981) diluted in PBS containing 1% BSA overnight at 4 °C, followed by addition of horseradish peroxidase-coupled secondary antibodies and DAB colorimetric reaction. The specimens were then processed routinely by washing, dehydration, and mounting. Peroxidase-labeled specimens were observed in a Nikon light microscope equipped with Polychrome-III camera (YC technology, New Taipei City, Taiwan) and Image Eye software (FMJ Software, Stockholm, Sweden).

### 2,3,5-Triphenyltetrazolium chloride (TTC) staining

Briefly, 5–7 brain slices (2 mm) were made from the olfactory bulb to the cerebellum using a razor blade. Slices were incubated in freshly prepared 2% 2,3,5 triphenyltetrazolium chloride (TTC) in PBS for 30 min. Images of the stained brain sections were captured with a digital camera.

### Statistical analysis

All data were expressed as the mean ± SEM from at least 3 independent experiments (*N* ≥ 3). The significance of the variation between the control and experimental test condition was analyzed by Student’s t-test. The significance of the difference among the groups was determined using One-Way Analysis of Variance (ANOVA), followed by Duncan’s method. *p* < 0.05 was considered statistically significant.

## Supplementary information

**Additional file 1: Table S1.** Characteristics of gold particles. **Table S2.** Effects of Au-NPs and Au-MPs on bEnd.3 cell viability. **Table S3.** Primer sets for qPCR. **Figure S1.** Changes in mRNA level of AQP1 and Cav1 in Au-NP-treated bEnd.3 cells. **Figure S2.** Au-NPs did not induce AQP1 expression in cells with hypo-expression level of Cav1. **Figure S3.** Au-NP treatment rapidly changed the phosphorylating status of FAK, AKT and ERK in concentration-dependent manner. **Figure S4.** The ERKs functioned as the negative controller on Cav1 and AQP1 expression in bEnd.3 cells. **Figure S5.** Proposed signaling pathway responsible for Au-NP-mediated AQP1 expression in bEnd.3 endothelial cells.

## Data Availability

The datasets used and/or analysed during the current study are available from the corresponding author on reasonable request.

## Abbreviations

AQP1: Aquaporin 1
Au-MPs: Gold microparticles
Au-NPs: Gold nanoparticles
BBB: Blood-brain barrier
Cav1: Caveolin 1
ERK: Extracellular regulated protein kinase
FAK: Focal adhesion kinase
