# Correction to: Inhaled gold nanoparticles cause cerebral edema and upregulate endothelial aquaporin 1 expression, involving caveolin 1 dependent repression of extracellular regulated protein kinase activity

**DOI:** 10.1186/s12989-019-0329-x

**Published:** 2019-11-18

**Authors:** Ching-Yi Chen, Po-Lin Liao, Chi-Hao Tsai, Yen-Ju Chan, Yu-Wen Cheng, Ling-Ling Hwang, Kuan-Hung Lin, Ting-Ling Yen, Ching-Hao Li

**Affiliations:** 1 Department of Physiology, School of Medicine, College of Medicine, Taipei Medical University, 250 Wuxing Street, Taipei, 110 Taiwan; 2 Graduate Institute of Medical Sciences, College of Medicine, Taipei Medical University, Taipei, Taiwan; 3 School of Pharmacy, Taipei Medical University, Taipei, Taiwan; 4 Institute of Food Safety and Health Risk Assessment, School of Pharmaceutical Sciences, National Yang-Ming University, Taipei, Taiwan; 5 Institute of Biomedical Sciences, Mackay Medical College, New Taipei City, Taiwan; 6 Department of Medical Research, Cathay General Hospital, Taipei, 22174 Taiwan

**Correction to: Part Fibre Toxicol**

**https://doi.org/10.1186/s12989-019-0324-2**

It was highlighted that the original article [1] contained the wrong Fig. 1. This Correction article shows the correct Fig. 1. The original article has been updated.

**Fig. 1:**
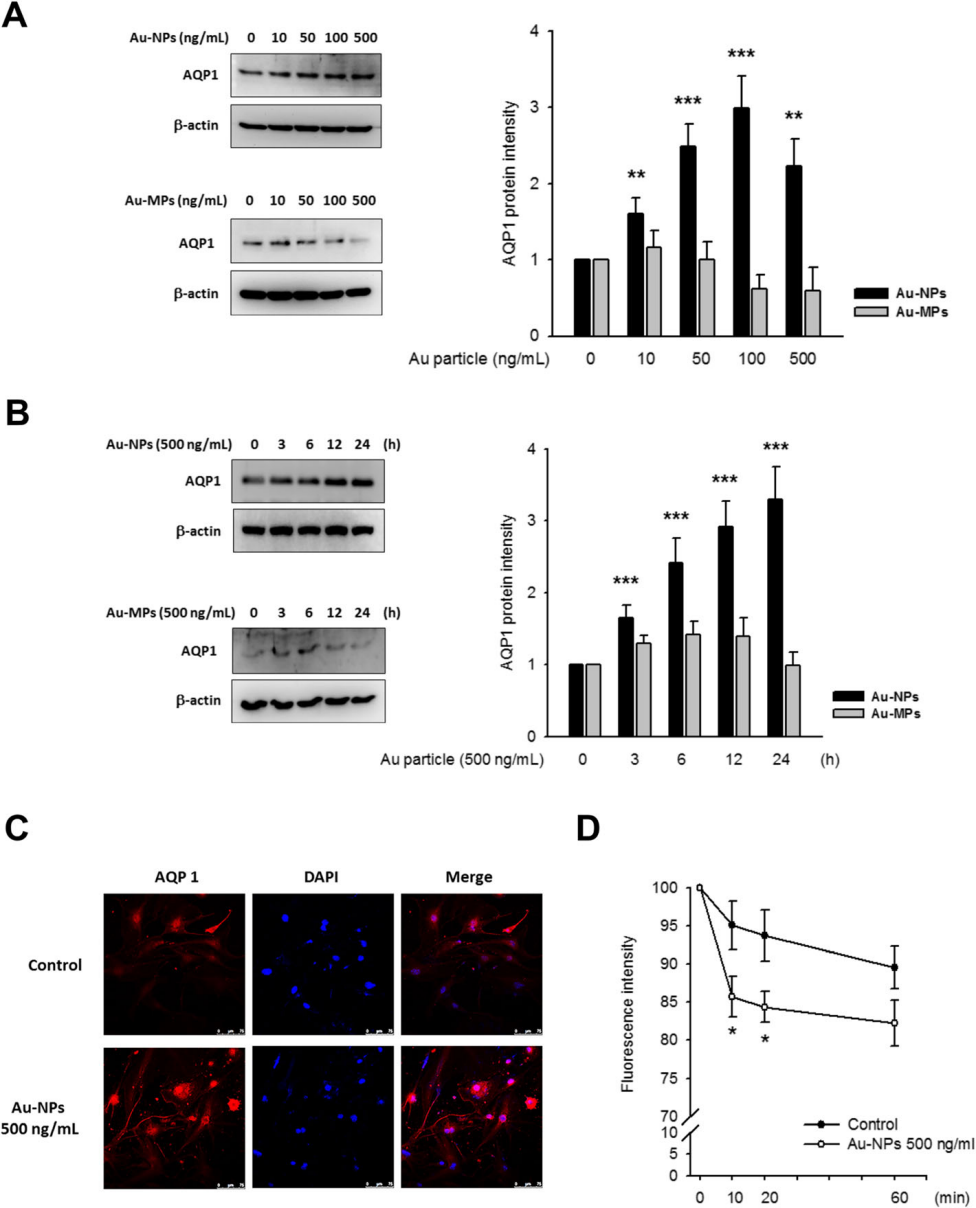
Au-NPs induced aquaporin-1 (AQP1) protein expression in bEnd.3 cells. **a**/**b** The bEnd.3 cells (an immortalized mouse cerebral endothelial cell line) were exposed to Au-NPs (or Au-MPs) and the expression level of AQP1 was detected by western blots. Representative images showed an increase of AQP1 protein level in Au-NP-treated groups, whereas AQP1 protein level remained unaffected in Au-MP-treated groups. **a** concentration-dependent treatment; cells were incubated with 10, 50, 100 and 500 ng/mL Au-NPs for 24 h. **b** time-dependent treatment; cells were incubated with 500 ng/mL Au-NPs for 3, 6, 12, and 24 h. (* *p* < 0.05, ** *p* < 0.01, and *** *p* < 0.001 indicates statistically significant difference from the control group; *N* = 11). **c** Representative images of immunofluorescent staining, the Au-NP-induced AQP1 and the nucleus was manifested by red and blue fluorescence, respectively. A gain of red fluorescence in cell membrane and cytosol was observed in Au-NP-treated bEnd.3 cells (500 ng/mL; 24 h), as compared to control. **d** Transendothelial permeability assay was performed as described in Materials and Methods. Au-NP treatment (500 ng/mL; 24 h) made bEnd.3 cell more permeable to water. (* *p* < 0.05, indicates statistically significant difference from the control group; *N* = 12)
