# Supplementary material for: Inhaled gold nanoparticles cause cerebral edema and upregulate endothelial aquaporin 1 expression, involving caveolin 1 dependent repression of extracellular regulated protein kinase activity
Source: Part Fibre Toxicol. 2019 Oct 16;16:37. doi: 10.1186/s12989-019-0324-2 (PMC6796418; doi:10.1186/s12989-019-0324-2)
Supplement: Supplementary file 1 — Additional file 1: Table S1. Characteristics of gold particles. Table S2. Effects of Au-NPs and Au-MPs on bEnd.3 cell viability. Table S3. Primer sets for qPCR. Figure S1. Changes in mRNA level of AQP1 and Cav1 in Au-NP-treated bEnd.3 cells. Figure S2. Au-NPs did not induce AQP1 expression in cells with hypo-expression level of Cav1. Figure S3. Au-NP treatment rapidly changed the phosphorylating status of FAK, AKT and ERK in concentration-dependent manner. Figure S4. The ERKs functioned as the negative controller on Cav1 and AQP1 expression in bEnd.3 cells. Figure S5. Proposed signaling pathway responsible for Au-NP-mediated AQP1 expression in bEnd.3 endothelial cells. [file 12989_2019_324_MOESM1_ESM.docx]

**Supplementary Information**

**Table S1. Characteristics of gold particles.**

|  | **Characterization of gold particles** | |
| --- | --- | --- |
|  | **Au-NPs** | **Au-MPs** |
| **TEM image** | 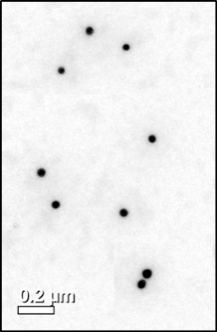 | 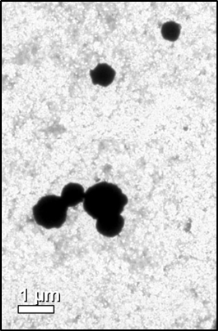 |
| **Mean primary particle size (nm) (TEM)** | 40 ± 1 | 637 ± 9 |
| **Hydrodynamic diameter (nm)** | 151 ± 18 | 480 ± 180 |
| **Zeta potential (mV)** | −10.7 ± 1.5 | −22.2 ± 6.5 |

1. For further information, please refer to our earlier publication.**^[7]^**
2. Optical property of Au-NPs was identified by the absorption spectra at the wavelength 500-550 nm.
3. Data are expressed as means ± S.E.M.

**Table S2. Effects of Au-NPs and Au-MPs on bEnd.3 cell viability.**

**Table S2-1.**

| **Au-NPs (ng/mL)** | **Cell viability (%)** | | | |
| --- | --- | --- | --- | --- |
|  | **60 min treatment** | | **24 h treatment** | |
|  | **bEnd.3** | **bEnd.3 Cav1-KD** | **bEnd.3** | **bEnd.3 Cav1-KD** |
| **0** | 100.00 ± 0.00 | 100.00 ± 0.00 | 100.00 ± 0.00 | 100.00 ± 0.00 |
| **10** | 102.06 ± 2.96 | 100.84 ± 0.69 | 101.05 ± 2.60 | 99.51 ± 1.88 |
| **50** | 102.03 ± 2.83 | 100.83 ± 1.69 | 101.02 ± 3.56 | 97.85 ± 1.78 |
| **100** | 100.84 ± 1.56 | 101.96 ± 0.40 | 99.89 ± 4.13 | 98.01 ± 1.28 |
| **500** | 100.93 ± 3.77 | 100.25 ± 0.53 | 97.20 ± 13.60 | 97.96 ± 0.75 |

**Table S2-2.**

|  | **Cell viability (%)** |
| --- | --- |
| **Au-MPs (ng/mL)** | **bEnd.3** |
| **0** | 100.00±0.00 |
| **10** | 102.47±2.96 |
| **50** | 100.56±5.00 |
| **100** | 102.39±5.14 |
| **500** | 100.87±6.17 |

1. Cell viability was evaluated using the MTT assay. Briefly, bEnd.3 cells were treated with series of concentrations of Au-NPs or Au-MPs for 24 h or 60 min. After incubation, MTT reagent was added to the cultures, and cells were incubated at 37°C for another 2 h. Finally, the medium was gently removed, and the formazan was dissolved in DMSO. Absorption was measured at 570 nm.
2. Data are expressed as means ± S.D. (N = 5).
3. Treatment with Au-NPs exerted no clear cytotoxicity on bEnd.3 cells.

**Table S3. Primer sets for qPCR**

| Gene | Sense primer | Antisense primer |
| --- | --- | --- |
| mAQP1 | CTGCTGGCGATTGACTACACTG | GGTTTGAGAAGTTGCGGGTGAG |
| mCav1 | GACCCCAAGCATCTCAACGA | GCCATTGGGATGCCGAAGA |
| mActin | TGTCCACCTTCCAGCAGATGT | AGCTCAGTAACAGTCCGCCTAG |

**Figure S1.**


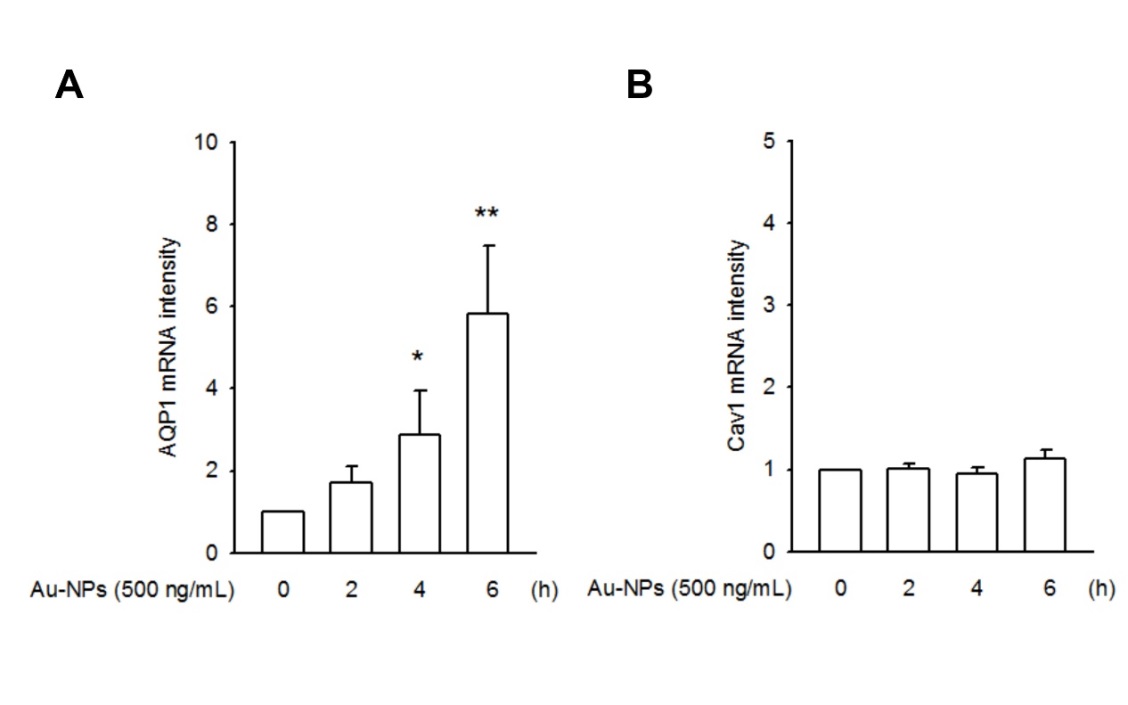


**Figure S1. Changes in mRNA level of AQP1 and Cav1 in Au-NP-treated bEnd.3 cells.**  We found that AQP1 was up-regulated in its mRNA level after 4-6 h Au-NP (500 ng/ml) treatment (A), whereas the expressing level of Cav1 was remained unaltered (B). (* *p* < 0.05, ** *p* < 0.01, indicates statistically significant difference from the control treatment; N = 5).

**Figure S2.**

**
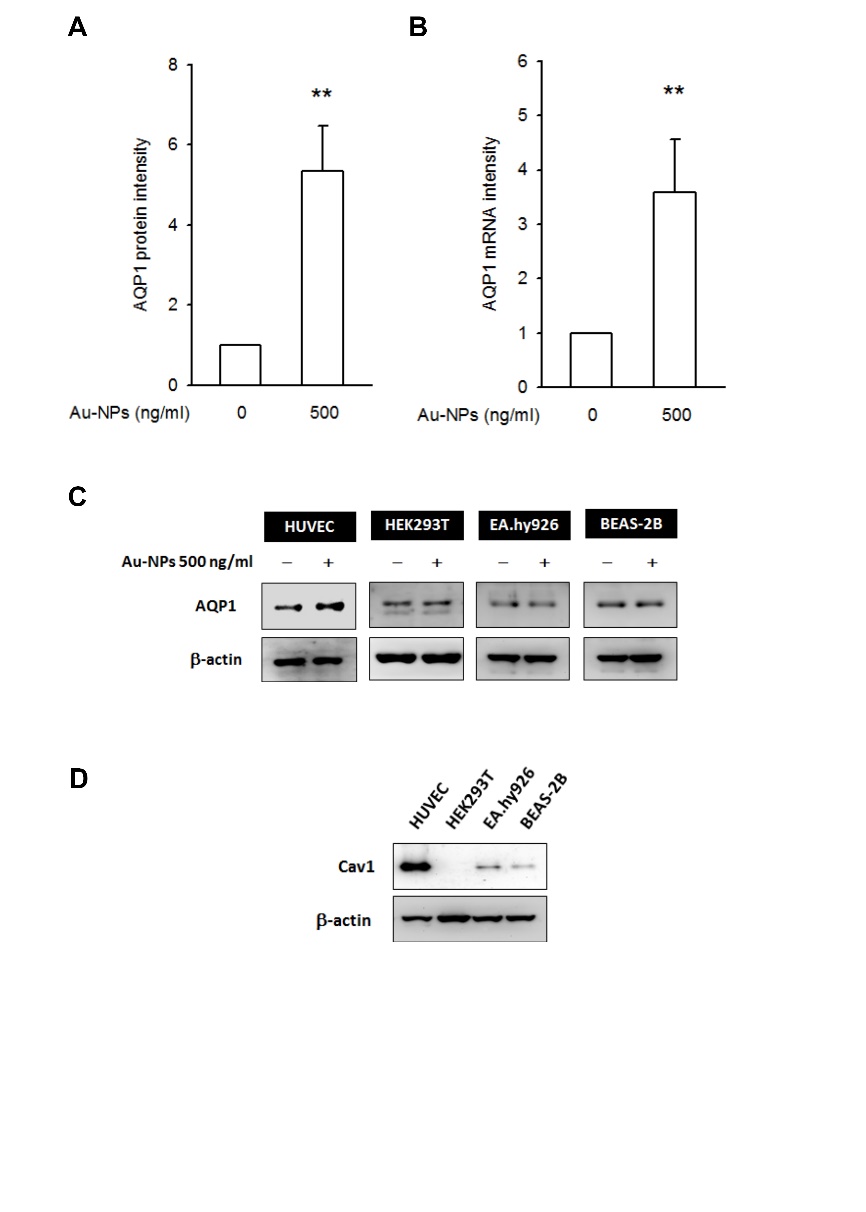
**

**Figure S2.** **Au-NPs did not induce AQP1 expression in cells with hypo-expression level of Cav1.** (A/B) Au-NP-induced AQP1 expression was presented in human umbilical vein endothelial cells (HUVEC), either in transcriptional level (N = 10) or in translational level (N = 5). (** *p* < 0.01, indicates statistically significant difference from the control group). (C/D) As compared to those cells with a hypo-expressing Cav1 (such as HEK293T, EA.hy926, and BEAS-2B), the HUVECs were abundant in its Cav1 protein level and responded to Au-NP treatment. No Au-NP-induced AQP1 expression were found in HEK293T (human embryonic kidney cell), EA.hy926 (a permanent hybrid cell line established by fusing HUVEC with A549), and BEAS-2B (an immortalized, normal human bronchial epithelial cell line).

**Figure S3.**

**
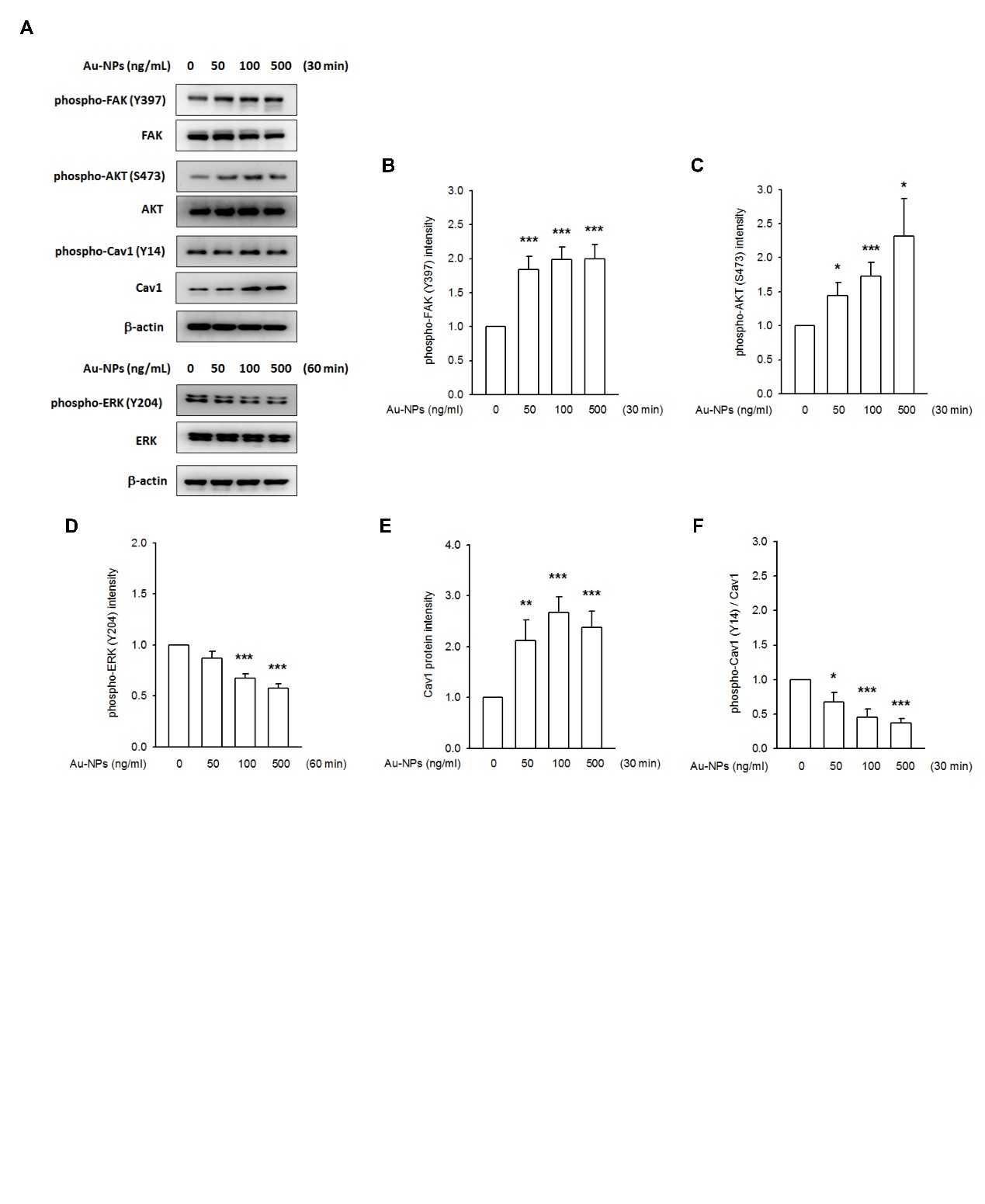
**

**Figure S3. Au-NP treatment rapidly changed the phosphorylating status of FAK, AKT and ERK in concentration-dependent manner.** bEnd.3 cells were incubated with Au-NPs (50, 100, 500 ng/mL) for 30 or 60 min. (A) Representative images showed an augmentation of FAK and AKT phosphorylation; an accumulation of Cav1 protein, but a reduced Cav1 phosphorylation level, after 30 min Au-NP treatment. A reduction of ERK activity was found after 60 min Au-NP treatment. Quantified data was gained by densitometry analysis, followed by a normalized process to their total form. (B) phospho-FAK, (C) phospho-AKT, (D) phospho-ERK, (E) Cav1 and (F) phospho-Cav1. (* *p* < 0.05, ** *p* < 0.01, and *** *p* < 0.001 indicates statistically significant difference from the control group; N > 7).

**Figure S4.**

**
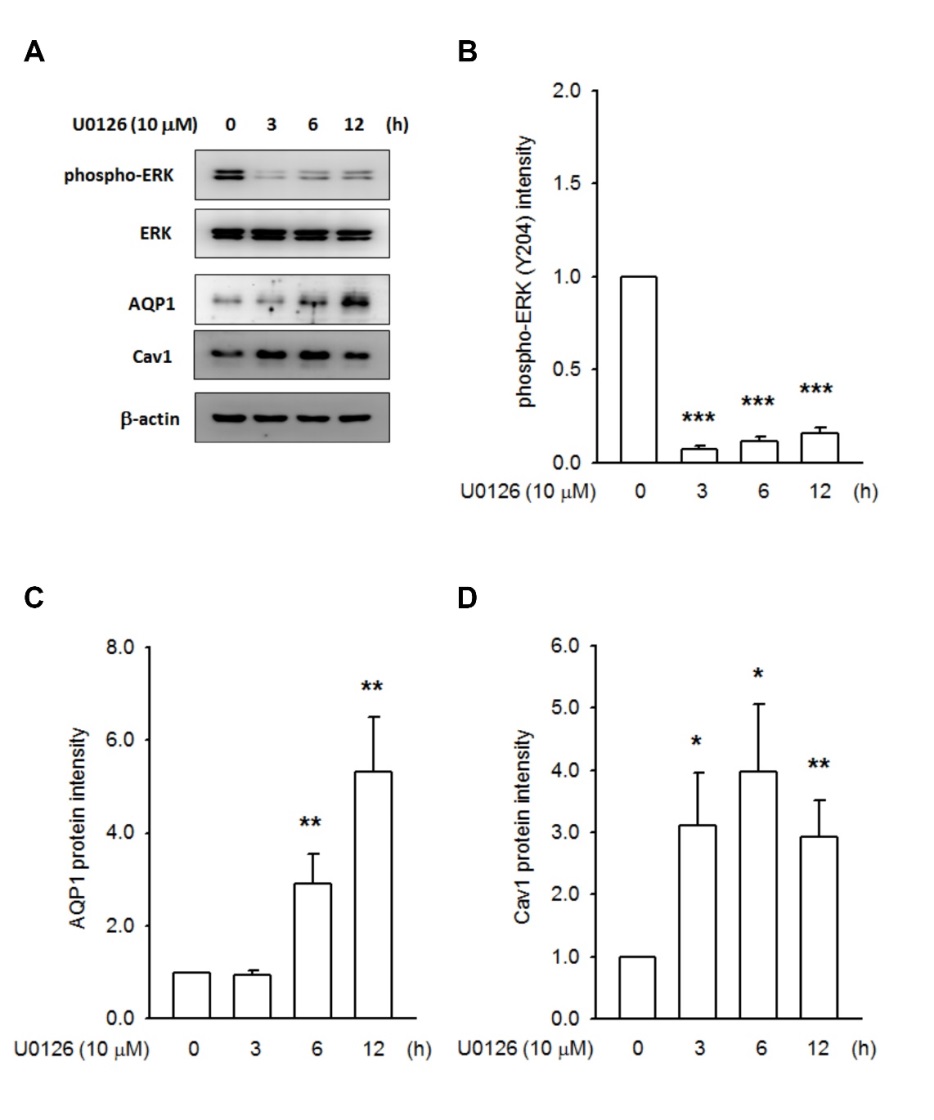
**

**Figure S4.** **The ERKs functioned as the negative controller on Cav1 and AQP1 expression in bEnd.3 cells.** bEnd.3 cells were incubated with ERK inhibitor, U0126 (10 μM) for 0, 3, 6 and 12 h. (A) Representative images showed the repression on ERK activity. A remarkable accumulation of Cav1 protein was observed after 3 h incubation, and sustained at least 12 h. In parallel, the AQP1 was increased after 6 h incubation. Quantified data was gained by densitometry analysis, followed by a normalized process to their total form or β-actin. (B) phospho-ERK, (C) AQP1, and (D) Cav1. (* *p* < 0.05, ** *p* < 0.01, and *** *p* < 0.001 indicates statistically significant difference from the control group; N = 4).

**Figure S5.**


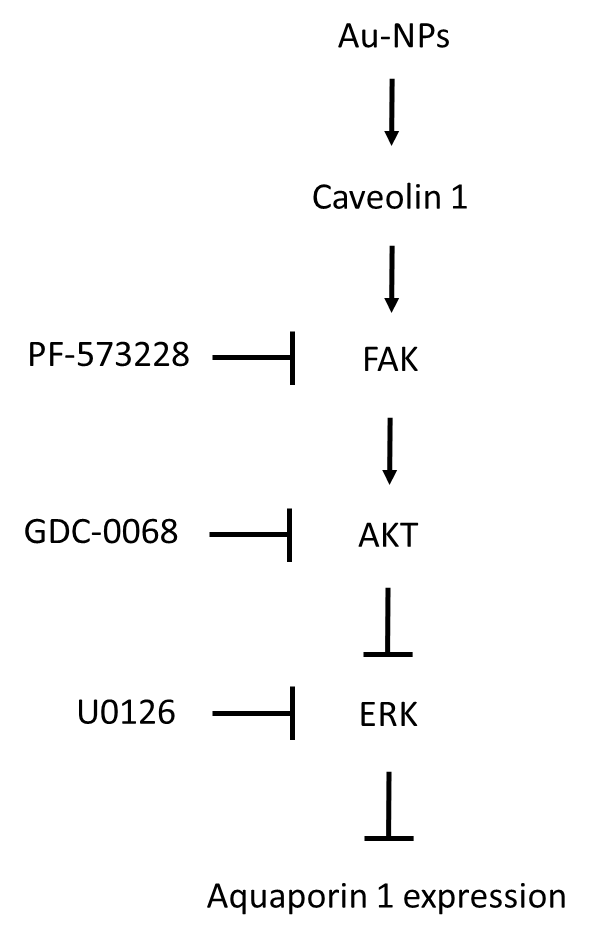


**Figure S5.** **Proposed signaling pathway responsible for Au-NP-mediated AQP1 expression in bEnd.3 endothelial cells** Au-NP exposure rapidly increased the Cav1 protein level, boosted the phosphorylation levels of FAK and AKT (after 15-30 min treatment), and reduced phospho-ERK level (after 60 min treatment). Neither these signaling molecules nor AQP1 expression responded to Au-NPs while Cav1 was silenced. The inhibition of AKT (GDC-0068) or FAK (PF-573228) has no obvious influence on Au-NP-mediated Cav1 accumulation, but rescued Au-NP-mediated ERK de-phosphorylation and prevented AQP1 induction. PF-573228 decreased the phospho-AKT level induced by Au-NPs, whereas GDC-0068 failed to prevent Au-NP-induced FAK phosphorylation, suggested FAK may be an upstream effector of AKT. Inhibition of ERK activity (U0126) remarkably enhanced AQP1 expression in bEnd.3 cells. These data demonstrate that Au-NP-mediated AQP1 induction is Cav1 dependent, but requires the repression on ERK activity.
